# Risk Factors for Distant Metastasis in Patients with Minimally Invasive Follicular Thyroid Carcinoma

**DOI:** 10.1371/journal.pone.0155489

**Published:** 2016-05-12

**Authors:** Yu-Mi Lee, Dong Eun Song, Tae Yong Kim, Tae-Yon Sung, Jong Ho Yoon, Ki-Wook Chung, Suck Joon Hong

**Affiliations:** 1 Department of Surgery, Asan Medical Center, University of Ulsan College of Medicine, Seoul, Korea; 2 Department of Pathology, Asan Medical Center, University of Ulsan College of Medicine, Seoul, Korea; 3 Department of Internal Medicine, Asan Medical Center, University of Ulsan College of Medicine, Seoul, Korea; Seoul National University, REPUBLIC OF KOREA

## Abstract

**Background:**

Although patients with minimally invasive follicular thyroid carcinoma (MIFTC) generally have an excellent prognosis, distant metastasis occurs in some patients. Risk factors for distant metastasis have been reported, none has been found to be conclusive. This study evaluated risk factors for distant metastasis, including protein markers, in patients with MIFTC.

**Methods:**

A review of patient records identified 259 patients who underwent surgery at Asan Medical Center from 1996 to 2010 and were subsequently diagnosed with MIFTC. After review of pathological slides, 120 patients with paraffin blocks suited for tissue microarrays (TMA) were included in this study. Immunohistochemical stain of TMA slides was performed by protein markers; β-catenin, C-MET, CK19, estrogen receptor (ER) α, ER β, HBME-1, MMP2, PPAR γ and progesterone receptor.

**Results:**

120 patients included 28 males (23.3%) and 92 females (76.7%), of mean age 41.5±10.8 years (range, 13–74 years). Eight patients (6.7%) had distant metastases during follow-up. Univariate analysis showed that age (≥45 years), male sex, and extensive vascular invasion (≥4 foci) were associated with distant metastasis. Multivariate regression analysis showed that extensive vascular invasion was the only independent risk factor for distant metastasis (p = 0.012). Although no protein markers on TMA analysis were directly related to distant metastasis of MIFTC, CK19 expression was more frequent in patients with than without extensive vascular invasion (p = 0.036).

**Conclusion:**

Extensive vascular invasion was the only independent risk factor for distant metastasis of MIFTC. No proteins markers were directly related to distant metastasis, but CK19 was associated with extensive vascular invasion.

## Introduction

Follicular thyroid carcinoma (FTC) is the second most common type of thyroid cancer, being present in 10–15% of patients with thyroid cancer. FTC can be histologically classified into two categories: minimally and widely invasive FTC. Minimally invasive FTC (MIFTC) is a grossly encapsulated solitary tumor with limited capsular and/or vascular invasion, whereas widely invasive FTC (WIFTC) is characterized by widespread infiltration of adjacent thyroid tissue and/or blood vessels by World Health Organization (WHO) classification. [1] Patients with MIFTC have an excellent prognosis because distant metastasis is very rare; by contrast, distant metastasis is observed in 10–30% of patients with WIFTC. [2, 3]

MIFTC is confirmed on pathological examination only after diagnostic hemithyroidectomy and it is difficult to preoperatively determine whether patients require complete thyroidectomy. Because of its excellent prognosis, it is generally known that MIFTCs do not need completion thyroidectomy. [2, 4, 5] Some patients at risk of developing distant metastasis, however, require complete thyroidectomy and radioactive iodine (RAI) ablation. [6–9]

Studies have suggested that age, sex, tumor size, and/or vascular invasion are clinicopathological risk factors for distant metastasis of MIFTC. Those studies, however, had limitations, including the small numbers of patients with distant metastases. Thus, to date, risk factors for distant metastasis remain unclear and there has been a growing interest to discover risk factors of distant metastasis through molecular biological research.

The expression of certain proteins may be related to distant metastases of MIFTC. Identification of specific biomarkers may allow identifying patients at risk for distant metastasis and in need of complete thyroidectomy and RAI ablation. Protein markers may be assessed by immunohistochemical (IHC) analysis of tissue microarrays (TMA), which is performed using the operative specimen and is cheap and convenient. This study was designed to evaluate clinicopathological risk factors for distant metastasis in patients with MIFTC and to determine protein biomarkers associated with patient prognosis.

## Material and Methods

### Tumor samples and patient data

**Fig 1:**
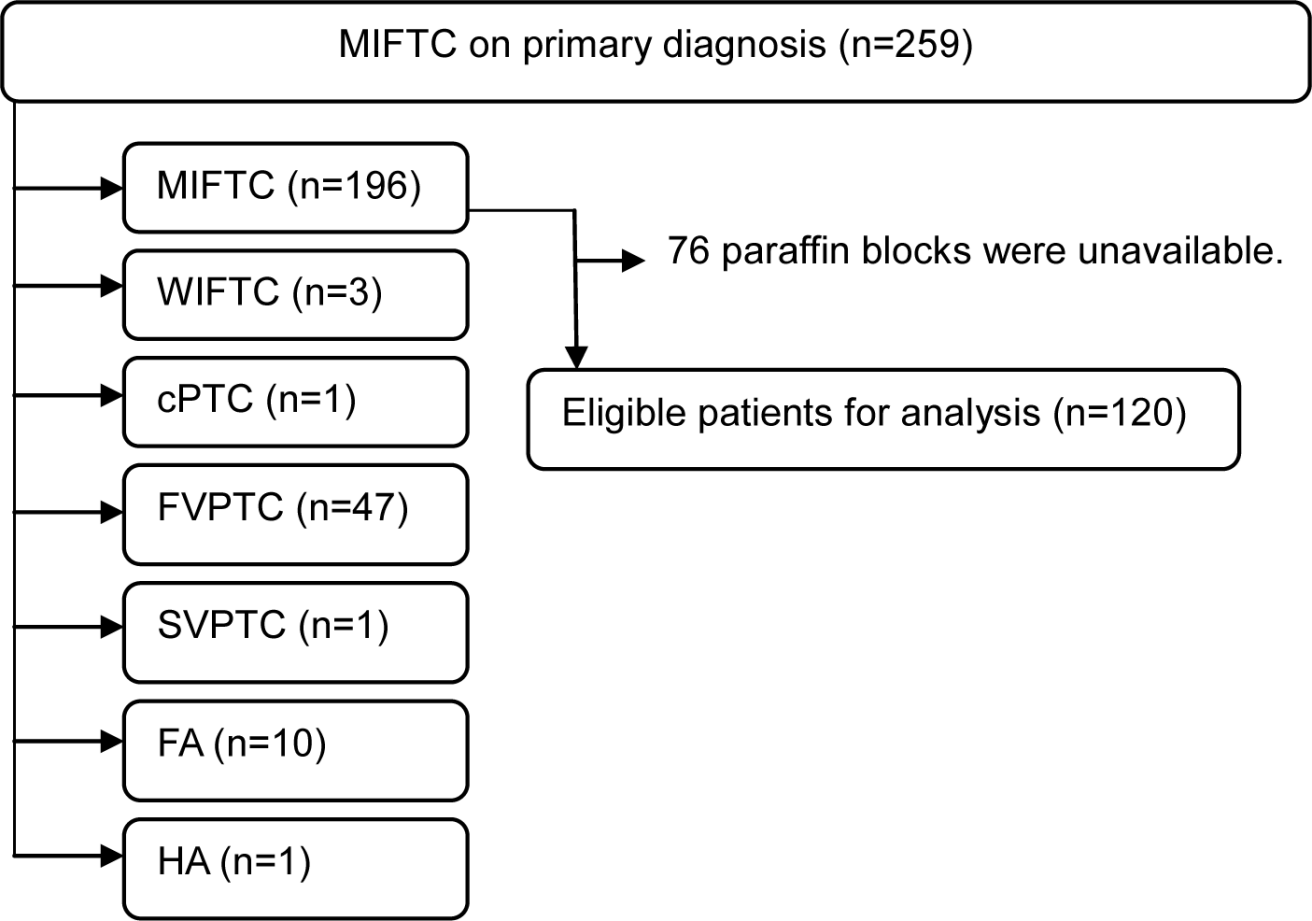
The algorithm of selection for eligible patients in this study. All H&E slides were reviewed by an experienced pathologist (DE Song). FA, follicular adenoma; cPTC, classical papillary thyroid carcinoma; FVPTC, follicular variant papillary thyroid carcinoma; HA, Hurthle cell adenoma; SVPTC; solid variant papillary thyroid carcinoma; WIFTC, widely invasive follicular thyroid carcinoma.

From February 1996 to December 2007, 259 patients were post-surgically diagnosed with MIFTC at Asan Medical Center. Hematoxylin and eosin (H&E) stained sections of these MIFTCs were reviewed according to WHO criteria by an experienced pathologist (DE Song). [1] 196 patients were confirmed as having MIFTC. Representative formalin-fixed and paraffin-embedded blocks were selected. Paraffin blocks for TMA were unavailable for 76 of these patients. Thus, 120 patients were included in this study (Fig 1).

The medical records of these patients were retrospectively reviewed, and their clinicopathological characteristics and outcome data were recorded. Written informed consent was not obtained from participants for their clinical records and tissues to be used in this study. Patient identifiers were removed from all samples and all the data were de-identified prior to analysis by a researcher who was irrelevant to this study. All experiments were conducted in accordance with the Declaration of Helsinki, and the study protocol was approved by the Institutional Review Board of Asan Medical Center.

### Construction of TMAs

Core tissue biopsies (2mm in diameter; three cores per patient) were taken from each paraffin block and arranged in new recipient paraffin blocks using TMArrayer (Beecher Instruments, Sun Prairie, WI, USA). One core was taken from the center of the tumor, and the other two cores were taken from the tumor borders. An additional core was taken from an area of normal thyroid as a control. Each block consisted of 54 cores, and a total of ten TMA blocks were produced. Sections from the TMA blocks were used for IHC stains in order to observe the expression of protein markers.

### Selection of candidate proteins and IHC analysis

Based on review of the literature, protein markers associated with tumor development, progression, and metastasis were selected, especially those previously identified in thyroid carcinoma. Antibodies of protein markers used in this study are summarized in Table 1.

**Table 1:** Antibodies and conditions used in the study.

Antibody	Clone	Manufacturer	Dilution
**β-catenin**	Polyclonal	Pharmingen, NJ	1: 500
**C-MET**	SP44	Ventana, Tusan	1: 4
**CK19**	A53-B/A2.26	Cell Marque, CA	1: 100
**ER α**	Polyclonal	Santacruz, Ca	1: 400
**ER β**	Polyclonal	Santacruz, Ca	1: 400
**HBME-1**	Polyclonal	DAKO, Denmark	1: 200
**MMP2**	Polyclonal	Santacruz, Ca	1: 10
**PPAR γ**	Polyclonal	Santacruz, Ca	1: 20
**PR**	16	NOVO, UK	1: 200

ER, estrogen receptor; PR, progesterone receptor

**Fig 2:**
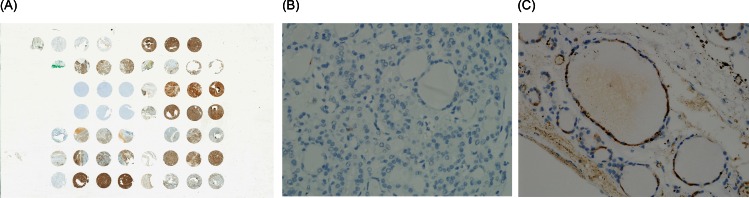
CK expression in minimally invasive follicular thyroid carcinoma. (A) Overview image of TMA slide stained by CK19, (B) negative staining and (C) positive staining for CK19 (immunohistochemistry, original magnification ×200).

IHC was performed using 4μm sections and the appropriate primary antibodies, with the slides being analyzed by an experienced pathologist (DE Song). Immunoreactivity was defined as positive if >10% of tumor cells showed unequivocal positive staining. All results were evaluated relative to a negative control from the same tissue sample (Fig 2).

### Validation

Although TMA can analyze many samples simultaneously, as well as analyze many types of markers, TMA has a disadvantage in that it represents a small part of each tumor. A validation test was performed to compensate for this disadvantage. Unstained slides produced from the original paraffin blocks of the entire tumors of all 120 MIFTCs were stained again. For validation staining, we only used antibodies of protein markers which showed relationship with MIFTC in TMA analysis. These slides were analyzed by the same experienced pathologist (DE Song), with immunoreactivity defined as above.

### Statistical analysis

Patients were classified into two groups, those with and without distant metastasis. Clinicopathological characteristics and expression of protein markers were compared in these two groups. In addition, the correlations between clinicopathological characteristics and expression of protein markers were analyzed.

Risk factors for recurrence were evaluated by univariate and multivariate Cox regression analysis. Recurrence-free survival was assessed by the Kaplan–Meier method and compared by the log rank test. The correlations between frequencies of expression of protein markers and the risk factors were analyzed by the χ^2^test or Fisher’s exact test. P values <0.05 were considered statistically significant. All statistical analyses were performed using the SPSS program, version 21.0 (SPSS Inc, Chicago, IL, USA)

## Results

### Clinicopathological characteristics related to distant metastasis

Of the 120 patients, eight (6.7%) developed distant metastasis during the follow-up period. The median follow-up period was 99.5 months (range, 13–222) and the median time to distant metastasis was 34.5 months (range, 13–133 months). The clinicopathological characteristics of these patients are summarized in Table 2.

**Table 2:** Clinicopathologic characteristics of patients with distant metastasis.

No.	Age (year)	Sex	Surgery	Tumor Size (cm)	Capsular invasion (foci)	Angioinvasion (foci)	Disease-free survival (months)	Distant metastasis	Treatment for metastasis	Status
**1**	57	Female	Hemithyroidectomy	4.5	0	10	100	Lung, bone	RAI, Surgery	Dead
**2**	54	Female	Total thyroidectomy	5.0	5	6	127	Lung, bone	RAI	Alive
**3**	57	Male	Total thyroidectomy	9.3	4	10	21	Lung, bone	RAI	Dead
**4**	51	Male	Hemithyroidectomy	0.8	0	2	25	Lung	RAI	Alive
**5**	47	Female	Hemithyroidectomy	2.7	1	0	133	Bone	RAI	Alive
**6**	53	Male	Total thyroidectomy	4	0	4	96	Lung	No	Alive
**7**	61	Male	Total thyroidectomy	5.2	1	4	19	Bone	RAI	Alive
**8**	27	Male	Total thyroidectomy	6.0	2	10	20	Bone	RAI	Alive

RAI, radioactive iodine

Age (≥45 years), male sex, tumor size ≥4.0 cm, undergoing total thyroidectomy, and extensive (≥4 foci) capsular or vascular invasion were more frequent in patients who did than did not develop distant metastasis. Univariate analysis showed that age (≥45 years; odds ratio [OR] = 5.07, 95% confidence interval [CI] = 1.01–25.43, p = 0.048), male sex (OR = 4.44, 95% CI = 1.08–18.25, p = 0.039), and extensive vascular invasion (OR = 6.86, 95% CI = 1.64–28.74, p = 0.008) were significant risk factors for distant metastasis of MIFTC. Multivariate analysis showed that extensive vascular invasion was the only independent risk factor related to distant metastasis (OR = 6.44, 95% CI = 1.50–27.69, p = 0.012) (Table 3).

**Table 3:** Univariate and multivariate analysis of risk factors for distant metastasis.

Variables		No recurrence 112 (93.3%)	Distant metastasis 8 (6.7%)	Univariate		Multivariate*	
				OR (95% CI)	P	OR (95% CI)	P
**Sex**	**Female**	89 (79.5%)	3 (37.5%)	Ref.	0.039	Ref.	0.14
	**Male**	23 (20.8%)	5 (62.5%)	4.44 (1.08–18.25)		3.15 (0.68–14.63)	
**Age**	**< 45 years**	70 (62.5%)	1 (12.5%)	Ref.	0.048	Ref.	0.15
	**≥ 45 years**	42 (37.5%)	7 (87.5%)	5.07 (1.01–25.43)		3.55 (0.62–20.22)	
**Surgical extent**	**Hemithyroidectomy**	66 (58.9%)	3 (37.5%)	Ref.	0.14	NA	NA
	**Total thyroidectomy**	46 (41.1%)	5 (62.5%)	3.32 (0.67–16.49)			
**Tumor size (cm)**	**< 4.0 cm**	63 (56.3%)	2 (25.0%)	Ref.	0.09	NA	NA
	**≥ 4.0 cm**	49 (43.8%)	6 (75.0%)	3.99 (0.80–19.85)			
**Capsular invasion**	**< 4 foci**	103 (92.0%)	5 (62.5%)	Ref.	0.16	NA	NA
	**≥ 4 foci**	9 (8.0%)	2 (37.5%)	3.17 (0.64–15.74)			
**Angioinvasion**	**< 4 foci**	105 (93.7%)	2 (25.0%)	Ref.	0.008	Ref.	0.012
	**≥ 4 foci**	7 (6.3%)	6 (75.0%)	6.86 (1.64–28.74)		6.44 (1.50–27.69)	

*Multivariate analysis was performed using variables that showed statistically difference in univariate analysis.

CI, confidence interval; NA, not available; Ref., reference

**Fig 3:**
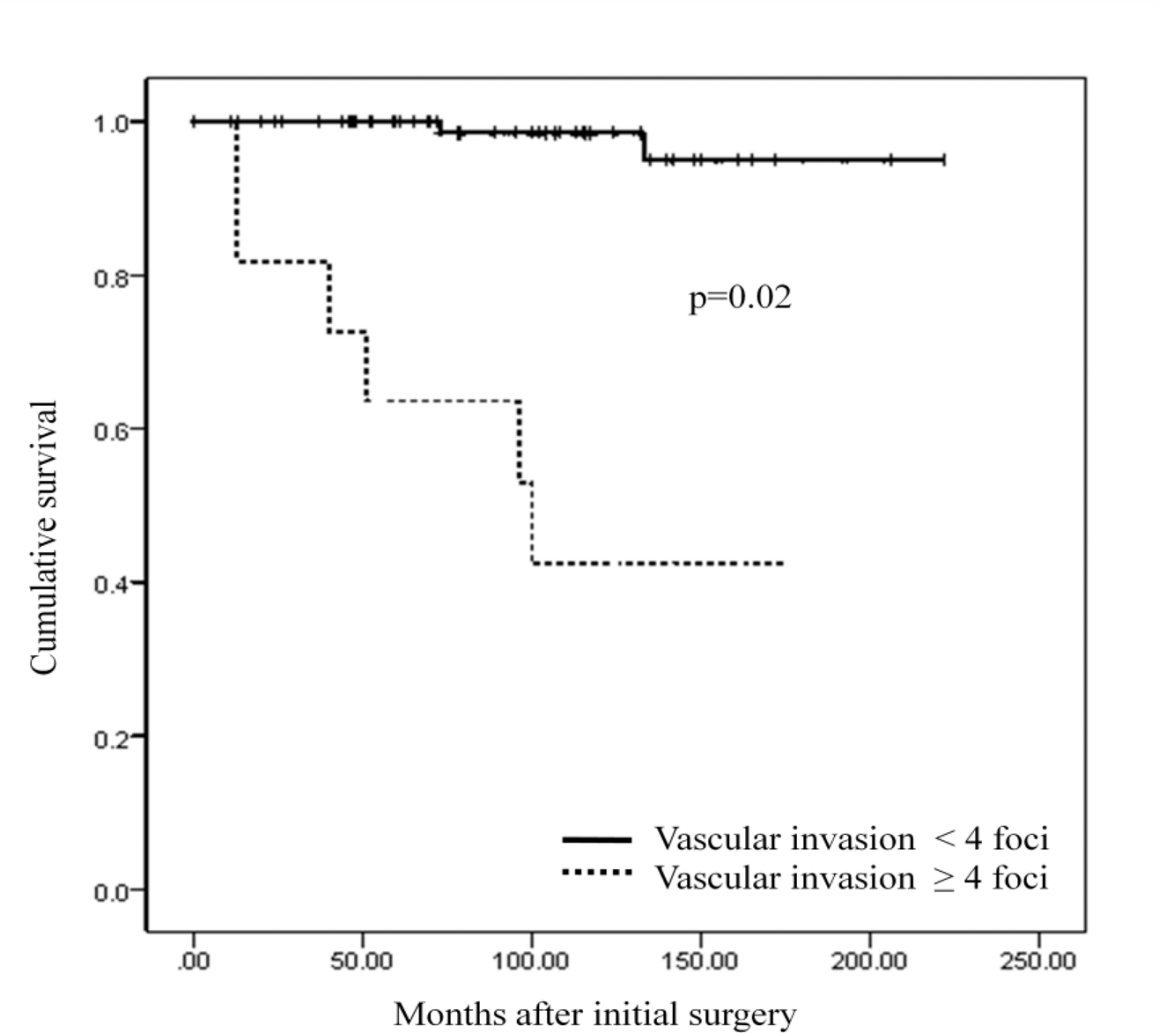
Comparison of recurrence free survival on basis of the presence of extensive vascular invasion.

Recurrence-free survival (RFS) was significantly shorter in patients with than without extensive vascular invasion (p = 0.02) (Fig 3). 5-year and 10-year RFS rates were 63.6% and 42.4% in patients with extensive vascular invasion, while 95.0% and 98.5% in patients without extensive vascular invasion.

### Protein markers associated with distant metastasis on TMA analysis

None of the protein markers assessed showed significant differences in expression between patients with and without distant metastasis (Table 4). Several protein markers, however, correlated with clinicopathological risk factors (Table 5). β-catenin and estrogen receptor α were more frequently expressed in male patients. CK19 was more frequently detected in patients with extensive vascular invasion.

**Table 4:** Comparison of the expression of protein markers between the group with distant metastasis and without distant metastasis.

	No recurrence (n = 112)	Distant metastasis (n = 8)	p
**β-catenin**	5 (4.5): 107 (95.5)	0 (0): 8 (100)	0.7
**C-MET**	3 (2.7): 109 (97.3)	0 (0): 8 (100)	0.81
**CK19**	41 (36.6): 71 (63.4)	4 (50.0): 4 (50.0)	0.35
**ER α**	4 (3.6): 108 (96.4)	0 (0): 8 (100)	0.76
**ER β**	42 (37.5): 70 (62.5)	4 (50.0): 4 (50.0)	0.36
**HBME-1**	75 (67.0): 37 (33.0)	6 (75.0): 2 (25.0)	0.49
**MMP2**	25 (22.3): 87 (77.7)	2 (25.0): 6 (75.0)	0.57
**PPAR γ**	32 (28.6): 80 (71.4)	3 (37.5): 5 (62.5)	0.43
**PR**	1 (0.9): 111 (99.1)	0 (0): 8 (100)	0.93

Data are described as positive (%): negative (%).

**Table 5:** Comparison of the expression of protein markers at TMA analysis according to clinicopathologic characteristics.

	Sex		p	Age (years)		p	Angioinvasion (foci)		p
	Male (n = 28)	Female (n = 92)		< 45 (n = 71)	≥ 45 (n = 49)		< 4 (n = 107)	≥ 4 (n = 13)	
**β-catenin**	3 (10.7)	2 (2.2)	0.048	2 (2.8)	3 (6.1)	0.33	5 (4.7)	0 (0)	0.56
**C-MET**	2 (7.1)	1 (1.1)	0.14	0 (0)	3 (6.1)	0.035	3 (2.8)	0 (0)	0.71
**CK19**	18 (64.3)	57 (62.0)	0.82	46 (64.8)	29 (59.2)	0.53	64 (59.8)	12 (92.3)	0.036
**ER α**	4 (14.3)	0 (0)	0.002	1 (1.4)	3 (6.1)	0.3	3 (2.8)	1 (7.7)	0.37
**ER β**	9 (32.1)	37 (40.2)	0.44	24 (33.8)	22 (44.9)	0.22	41 (38.3)	5 (38.5)	0.61
**HBME-1**	17 (60.7)	64 (69.6)	0.38	50 (70.4)	31 (63.3)	0.41	72 (67.3)	9 (69.2)	0.58
**MMP2**	8 (28.6)	19 (20.7)	0.38	15 (21.1)	12 (24.5)	0.67	22 (20.6)	5 (38.5)	0.14
**PPAR γ**	10 (35.7)	25 (27.2)	0.38	19 (26.8)	16 (32.7)	0.49	30 (28.0)	5 (38.5)	0.31
**PR**	0 (0)	1 (1.1)	0.77	1 (1.4)	0 (0)	0.59	1 (0.9)	0 (0)	0.89

Data are described as positive (%).

We only presented results with showing significant differences. The other clinicopathologic characteristics had no correlation with protein markers.

### Validation IHC staining test

Validation tests failed to identify any correlation between protein marker expression and the development of distant metastasis or the presence of clinicopathological risk factors (data not shown). Also, no protein markers showed a significant correlation when we performed analysis for validation between the expression of protein markers and clinicopathologic risk factors. CK19 expression was detected more frequently in patients with extensive vascular invasion, but its level of significance was borderline (p = 0.061).

## Discussion

The pathological diagnosis and treatment of FTC, especially MIFTC, is unclear due to the ambiguous criteria of the WHO classification. A review of all slides from patients initially diagnosed with MIFTC in our institution resulted in the reclassification of 63 of these patients as having other diagnoses, such as a follicular variant of papillary thyroid carcinoma or WIFTC. Although several proposals suggested that the criteria for FTC be revised, [2, 10, 11] no consensus has been reached on these criteria. All slides in this study were reviewed by a single experienced pathologist according to the WHO classification to prevent interobserver differences in diagnosis. The 120 patients confirmed as having MIFTC were included in this study.

This study found that extensive vascular invasion (≥4 foci) is an independent risk factor for distant metastasis in patients with MIFTC. Vascular invasion itself was reported to be a risk factor for distant metastasis of MIFTC, [2, 10, 12, 13] although other studies found that the mere presence of vascular invasion was not associated with prognosis. [5, 7, 8, 14] A review of 282 patients with MIFTC found that vascular invasion by four or more foci was associated with distant metastasis, suggesting the need for aggressive treatment, including mandatory complete thyroidectomy. [8] Our results are consistent with these earlier findings. The presence of vascular invasion itself was not a risk factor for distant metastasis (data not shown), while extensive vascular invasion was related to develop distant metastasis. Therefore we assumed that completion thyroidectomy followed by RAI therapy should be considered when extensive vascular invasion is present in MIFTC after diagnostic lobectomy.

Several other prognostic indices have been proposed for MIFTC. Many studies found that male sex, older age, and/or large tumor size were associated with poor prognosis. [2, 5–8, 13, 14] This study showed that age and sex were significantly associated with distant metastasis only on univariate analysis. Although these risk factors may affect prognosis, extensive vascular invasion was the most powerful risk factor. The results of multivariate analysis, however, were limited by the small number of patients in this study with distant metastasis.

This study was unable to identify protein markers directly related to distant metastasis in patients with MIFTC. Loss of estrogen receptor β and NRAS mutation have been associated with poor prognosis in patients with FTC, but these studies included patients with WIFTC. [15, 16] To date, no biomarkers have been found to be related to prognosis in patients with MIFTC. Although this study showed that CK19, estrogen receptor β, and PPAR-γ were differently expressed in patients with and without distant metastasis, the differences were not statistically significant.

Assessment of the correlation between expression of protein markers and clinicopathological characteristics showed that CK19 was more frequently expressed in patients who had MIFTC with extensive vascular invasion. In as much as extensive vascular invasion was an independent risk factor for distant metastasis, CK19 expression was indirectly associated with poorer prognosis in patients with MIFTC.

CK19 is a low molecular weight cytokeratin belonging to a subgroup of cytoskeletal proteins. Immunochemical detection of CK19 has been reported as diagnostic of PTC. [17, 18] Recently, CK19 expression was reported to adversely affect prognosis in patients with PTC, [19, 20] but the mechanism linking CK19 to the progression of thyroid carcinoma has not been determined. CK19 may be an indicator of stem cell capabilities and may indicate manifestations of undifferentiated characteristics in differentiated tumors. [21, 22] Moreover, CK19 may affect tumor metastasis by promoting cell mobility or extracellular degradation. [23] We assume that CK19 expression may be associated with the infiltration or migration of cancer cells and may correlate with the development of distant metastasis in patients with MIFTC. Expression of CK19, along with the presence of extensive vascular invasion, may be used to determine treatment strategies in patients with MIFTC invasion.

This study had several limitations. First, the number of patients who developed distant metastasis during the follow-up period was relatively small, it was difficult to obtain statistical determinations of the association between clinicopathological features and prognosis. Second, many paraffin blocks were in too poor condition to construct TMAs, making it difficult to evaluate the expression of protein markers in these sections from TMA blocks. Also, the TMA method itself is limited. A TMA cannot represent all of the features of an entire tumor, since each sample is taken from part of a tumor. To compensate for this drawback, TMAs were constructed by collecting three cores from each tumor, and validation tests were performed using the original paraffin blocks containing the entire tumors.

## Conclusion

Extensive vascular invasion (≥4 foci) was an independent risk factor for distant metastasis in patients with MIFTC. Although no protein markers were directly related to distant metastasis, CK19 was more frequently expressed in patients with than without extensive vascular invasion.
